# Spontaneous and CRISPR/Cas9-induced mutation of the osmosensor histidine kinase of the canola pathogen *Leptosphaeria maculans*

**DOI:** 10.1186/s40694-017-0043-0

**Published:** 2017-12-16

**Authors:** Alexander Idnurm, Andrew S. Urquhart, Dinesh R. Vummadi, Steven Chang, Angela P. Van de Wouw, Francisco J. López-Ruiz

**Affiliations:** 1 School of BioSciences, University of Melbourne, Building 122, Parkville, VIC 3010 Australia; 2 Department of Environment and Agriculture, Centre for Crop and Disease Management, Curtin University, Bentley, WA 6102 Australia

**Keywords:** *Agrobacterium*-mediated transformation, Canola, Gene editing, HOG pathway

## Abstract

**Background:**

The dicarboximide fungicide iprodione has been used to combat blackleg disease of canola (*Brassica napus*), caused by the fungus *Leptosphaeria maculans*. For example, in Australia the fungicide was used in the late 1990s but is no longer registered for use against blackleg disease, and therefore the impact of iprodione on *L. maculans* has not been investigated.

**Results:**

Resistance to iprodione emerged spontaneously under in vitro conditions at high frequency. A basis for this resistance was mutations in the *hos1* gene that encodes a predicted osmosensing histidine kinase. While loss of the homologous histidine kinase in some fungi has deleterious effects on growth and pathogenicity, the *L. maculans* strains with the *hos1* gene mutated had reduced growth under high salt conditions, but were still capable of causing lesions on *B. napus*. The relative ease to isolate mutants with resistance to iprodione provided a method to develop and then optimize a CRISPR/Cas9 system for gene disruptions in *L. maculans*, a species that until now has been particularly difficult to manipulate by targeted gene disruptions.

**Conclusions:**

While iprodione is initially effective against *L. maculans* in vitro, resistance emerges easily and these strains are able to cause lesions on canola. This may explain the limited efficacy of iprodione in field conditions. Iprodione resistance, such as through mutations of genes like *hos1*, provides an effective direction for the optimization of gene disruption techniques.

**Electronic supplementary material:**

The online version of this article (10.1186/s40694-017-0043-0) contains supplementary material, which is available to authorized users.

## Background

Canola (*Brassica napus*) is a major crop worldwide, and is also grown as part of the crop rotation systems with cereals [1]. Control of the main disease of canola, blackleg, is through farming practices that minimize exposure to the infectious spores, sowing cultivars that carry resistance genes, and more recently relying on fungicides. Blackleg disease is caused by a species complex in the genus *Leptosphaeria* (Dothideomycetes; Pleosporales) [2–4], with most crop losses due to *L. maculans*. Although a number of molecular biology resources are available for *L. maculans*, including a genome sequence [5], investigations of gene functions in the fungus has been hampered by the low rates of homologous integration of constructs used in generating gene deletion strains, with just nine gene knock outs reported in the literature [6–11]. Hence, this aspect of the fungus has limited the ability to test how specific genes may impact the ability of this fungus to cause disease on canola.

The application of fungicides has provided large yield increases to crops by reducing the symptoms caused by fungal diseases. In the case of blackleg disease, different fungicides have been and continue to be employed [1]. In Australia, currently these are in the triazole class [Fungicide Resistance Action Committee (FRAC) group 3], and used as seed dressings, combined with fertilizer, or as a foliar spray. In another class, the dicarboximide iprodione (FRAC group 2; trade name Rovral^®^ produced by Bayer CropScience), was approved for use against blackleg disease and used for about 5 years before being unregistered at the end of the 1990s. Iprodione currently can still be used for treatment of Sclerotinia stem rot of canola, which is a disease that has an overlapping distribution as blackleg.

Resistance to dicarboximide fungicides, like iprodione, can occur if mutations arise in the high osmolarity glycerol response (HOG) pathway [12]. The HOG pathway was first characterized in *Saccharomyces cerevisiae* for its role in enabling growth under hyper- and hypo-osmotic conditions [13–15], and subsequent research in numerous fungal species has defined multiple phenotypes of strains with mutations in the signaling genes. The HOG pathway features a sensing histidine kinase that transfers environmental information into a cascade of three mitogen activated protein kinases [12]. Mutations often occur in the homologs of the histidine kinase in other fungi to confer resistance to the dicarboximide fungicides [16].

Exposure to fungicides is linked to the emergence of fungicide resistance, thereby rendering specific fungicides or entire classes ineffective. We recently initiated an investigation into the levels of resistance to triazole fungicides in *L. maculans* populations in Australia [17]. As a control, isolates were tested for their responses against the unrelated chemical iprodione. Spontaneous resistance to this molecule was commonly observed, leading to the investigation into its basis and the impact of those mutations on pathogenicity. Subsequently, the *hos1* gene that was mutated in these resistant strains was used as a tool for the development of the clustered regularly interspaced short palindromic repeats (CRISPR)-Cas9 system to make targeted mutations in *L. maculans*.

## Methods

### Fungal strains and culturing

Routine culturing of *L. maculans* was on 10% V8 juice with 2% agar. The wild type strains or those isolated during this study are listed in Table 1. Because there is often limited contrast between the color of the V8 juice and fungal hyphae, to increase the contrast in the figures the strains were cultured on potato dextrose agar. Iprodione was dissolved in dimethyl sulfoxide, and added to agar media at final concentrations of 5 or 10 µg/ml.

**Table 1:** Strains of *Leptosphaeria* spp. used in this study

Strain name(s)	Genotype	Origin
*L. biglobosa* 06J154	Wild type	Burren Junction, NSW, Australia, 2006 [19]
D5 (IBCN18; M1)	Wild type	Penshurst, VIC, Australia, 1988
D5-Ip^R^	*hos1* ^−^	Selection of D5 on iprodione
D5-Ip^R^ + *hos1*	*hos1* ^−^ + *hos1*	Transformation of D5-Ip^R^ with wild type *hos1*
D2 (IBCN15)	Wild type	Streatham, VIC, Australia, 1988 [2]
D2-Ip^R^	*hos1* ^−^	Selection of D2 on iprodione
D2-Ip^R^ + *hos1*	*hos1* ^−^ + *hos1*	Transformation of D2-Ip^R^ with wild type *hos1*
D3 (IBCN16)	Wild type	Mt Barker, WA, Australia, 1988 [2]
D3-Ip^R^	*hos1* ^−^	Selection of D3 on iprodione
D3-Ip^R^ + *hos1*	*hos1* ^−^ + *hos1*	Transformation of D3-Ip^R^ with wild type *hos1*
14P290	Wild type	Katanning, WA, Australia, 2014 [17]
14P290-Ip^R^	*hos1* ^−^	Selection of 14P290 on iprodione
14P290-Ip^R^ + *hos1*	*hos1* ^−^ + *hos1*	Transformation of 14P290-Ip^R^ with wild type *hos1*
D13 (09SMW024)	Wild type	Cummins, SA, Australia, 2009 [52]
DV1	*hos1 guide*	D5
DV2	*hos1 guide*; *cas9*	DV1
DV3	*hos1 guide*; *cas9*; *hos1* ^−^	DV2
D13-CoT	*cas9*; *hos1 guide*	D13 cotranformed with both guide RNA (hyg) and Cas9 (G418)
D13-Ip^R^1	*cas9*; *hos1 guide*; *hos1* ^−^	Selection of D13-CoT on iprodione
D13-Ip^R^2	*cas9*; *hos1 guide*; *hos1* ^−^	Selection of D13-CoT on iprodione
v23.1.3	Wild type	*In vitro* crosses, France [5]
JN3-Cas9	*cas9*	v23.1.3 transformed with pMAI23
JN3-avrLm1-1	*cas9*; *AvrLm1 guide*; *avrLm1* ^−^	JN3-Cas9
JN3-avrLm1-2	*cas9*; *AvrLm1 guide*; *avrLm1* ^−^	JN3-Cas9

All strains are *L. maculans*, with the exception of one *L. biglobosa* strain used as a source of DNA for constructs. The numerous *hos1* mutants isolated from CRISPR-Cas9 sources and the 28 progeny from the D3-Ip^R^ × D13 cross are not listed. IBCN indicates a strain in the International Blackleg of Crucifers Network collection

For genetic segregation analysis, crosses were set up between strain D3-Ip^R^ and strain D13, on 20% V8 juice and CaCO_3_ medium. After 1 week of growth, the plates were overlaid with water agar. Cultures were incubated at 14 °C with alternating 12 h dark-blacklight cycles for 6 weeks. At this point, the plates were examined for the formation of pseudothecia. Asci were released by placing the pseudothecia in sterile water whereby ascospores were discharged naturally. Individual ascospores were then collected and allowed to germinate on 2% water agar plates before being hyphal-tip subcultured to create individual strains. A total of 28 progeny was collected and analyzed.

### DNA isolation of *L. maculans*, PCR and sequencing

*Leptosphaeria maculans* mycelia were cultured in 10% cleared V8 juice medium (pH 6). Mycelia were freeze-dried, broken with 2 mm glass beads, and DNA extracted in a CTAB buffer and incubation at 65 °C, followed by one chloroform extraction, and precipitation with an equal volume of isopropanol [18].

For sequencing, *hos1* was amplified with different primer combinations to cover different regions of this large gene. Amplicons used to identify spontaneous mutations were MAI0218-MAI0223 and MAI0220-MAI0224. Primer sequences used in this study are found in Additional file 1: Table S1. Primers used to amplify and then identify mutations in *hos1* induced by CRISPR-Cas9 were MAI0220-MAI0224.

To resolve which *hos1* allele is present in progeny of the D3-Ip^R^ × D13 cross, the region was amplified with primers MAI0220-MAI0376. The DNA was precipitated and then digested with AgeI restriction enzyme, which cuts the amplicon of the wild type copy but not the iprodione resistance allele.

Primers used to amplify the *AvrLm1* gene to identify mutations induced by CRISPR-Cas9 were MAI0353-MAI0354.

### Construction of plasmids for transformation of *L. maculans*

Plasmids were constructed for the introduction of T-DNA molecules into *L. maculans* using *Agrobacterium tumefaciens* mediated transformation.

Two plasmids conferring resistance to G418 or hygromycin were made, in which gene expression was from the promoter and terminator of the actin gene of *L. biglobosa* strain 06J154 [19]. G418 resistance has not been used previously in *L. maculans* transformation. For the G418 construct, the promoter region was amplified with primers MAI0014-MAI0015, and terminator region with primers MAI0016-MAI0017 from genomic DNA of strain 06J154, isolated as for *L. maculans*. The open reading for the aminoglycoside phosphotransferase that confers resistance to G418 was amplified with primers ALID0835-ALID0836 from plasmid pPZP-NEO1 [20]. The three pieces were joined by overlap PCR using primers MAI0014-MAI0017, and cloned into the TOPO pCR2.1 plasmid (Invitrogen). To ensure the expression system worked for resistance to G418, the equivalent plasmid was made to confer resistance to hygromycin. The promoter was amplified with primers MAI0018-MAI0024 and terminator with MAI0020-MAI0021. The hygromycin phosphotransferase gene was amplified with primers MAI0022-MAI0023 from plasmid pPZPHyg*Hin*dX [21]. The three pieces were joined together using primers MAI0018-MAI0021, and cloned into the TOPO 4.0 plasmid (Invitrogen). All PCRs used Platinum^®^ *Pfx* DNA polymerase (Invitrogen). Plasmids containing clones without PCR-derived errors were identified, by sequencing the inserted fragments. The G418 resistance construct was excised with EcoRI and cloned into the EcoRI site of plasmid pPZP-201BK, which is able to replicate in *Agrobacterium tumefaciens* [22], to form pMAI2. The hygromycin resistance construct was excised with KpnI-SpeI and cloned into the KpnI-XbaI site of pPZP-201BK to form plasmid pMAI6.

Plasmid pLAU2 was constructed by cloning two fragments, the *L. maculans* actin (*act1*) promoter and the *trp3* terminator, into pPZPHyg*Hin*dX [21] digested with AscI and PacI using Gibson Assembly (New England Biolabs). Primers used to amplify the actin promoter, with Platinum^®^ *Pfx* DNA Polymerase (Invitrogen), were AU1 and AU2 and for the *trp3* terminator were AU5 and AU6. Primers were designed with additional nucleotides such that a BglII site was introduced between the promoter and terminator. Green fluorescent protein (GFP) was amplified with primers AU28 and AU31, and cloned into the BglII site of pLAU2 to form plasmid pLAU17. The *act1* promoter and *trp3* terminator combination was excised from plasmid pLAU2 using restriction enzymes SpeI and NheI and cloned into the XbaI site of plasmid pMAI2, to form pLAU53.

For complementation with the wild type copy of *hos1*, the gene was amplified with primers MAI0206 and MAI0207 using Q5 DNA polymerase (New England Biolabs) from genomic DNA of wild type isolate D5, and cloned using Gibson assembly (New England Biolabs) into plasmid pMAI2 that had been linearized with EcoRV and XhoI.

Constructs were made to express either the Cas9 endonuclease or the CRISPR guide RNAs using the actin promoter of *L. maculans*. Cas9 was amplified with primers MAI0225 and MAI0226 from plasmid pHSN401 [23] and cloned into the BglII sites of pLAU2 and pLAU53 by Gibson assembly to form plasmids pMAI22 and pMAI23, respectively. The DNA fragment to target the endonuclease to *hos1*, guide RNA and two ribozymes were synthesized by Thermo Fisher Scientific (sequence in Additional file 2) and provided as a cloned product. The fragment was amplified using primers MAI0228 and MAI0229 and then inserted into the BglII sites of both plasmids pLAU2 and pLAU53 using Gibson assembly.

To streamline the production of the guide RNAs, two additional plasmids were made that incorporate the hepatitis delta virus (HDV) ribozyme, such that a single oligonucleotide of about 100 nt can be used for cloning the targeting RNA, rather than a synthesized and cloned DNA fragment. A XhoI restriction enzyme site was included to facilitate subsequent cloning of the gene-specific fragments. A DNA molecule (Additional file 2) was synthesized by Thermo Fisher Scientific, and amplified with primers MAI0228-MAI0229 and cloned into the BglII sites of both plasmids pLAU2 and pLAU53 using Gibson assembly to form plasmids pMAI75 and pMAI97, respectively.

A construct to produce a guide RNA to target mutations to the *AvrLm1* gene was generated, by amplification off oligonucleotide MAI0336 with primers MAI0309-MAI0310, and cloning the amplicon into plasmid pMAI75 linearized with XhoI.

In all cases of plasmid construction that used amplification of DNA and subsequent cloning, the inserted DNA molecules in the plasmids were sequenced to either confirm that no PCR-induced errors occurred or to identify error-free clones.

### Transformation of *L. maculans* with *Agrobacterium tumefaciens*

The plasmids were transformed into *A. tumefaciens* strain EHA105 using electroporation, and selected on LB medium + kanamycin (50 µg/ml). The *Agrobacterium* strains were then used to transform strains of *L. maculans*, with selection of fungal transformants using either G418 (100 µg/ml) or hygromycin (50 µg/ml), and cefotaxime (100–150 µg/ml) to inhibit *Agrobacterium* growth. The transformation of *L. maculans* was as follows. Overnight cultures of *Agrobacterium* in LB + kanamycin were diluted in sterile water and plated onto *Agrobacterium* induction medium [24] solidified with 2% agar (25 ml in 15 cm diameter Petri dishes) with pycnidiospores harvested in sterilized water from the *L. maculans* strains. Bacterial and fungal cells were spread across the plate. After 3 days incubation at 22 °C in darkness, 25 ml of cleared V8 juice 1.5% agar media containing selective antibiotics were overlaid. Transformed colonies emerged through the overlay agar 10–18 days later. The transformed strains were subcultured at least once onto V8 juice agar supplemented with the antibiotic suitable to select for fungal transformation and cefotaxime to inhibit *Agrobacterium* growth.

### Quantification of antifungal drug resistance

Levels of resistance to two dicarboximide fungicides and a triazole fungicide were quantified in a radial growth assay, as in Ref. [25] with some modifications. In brief, for each strain 4 mm diameter mycelium plugs were inoculated into the center of 9-cm PDA petri dishes amended with a range of concentrations of technical grade iprodione (0.195–50 µg/ml), procymidone (0.195–50 µg/ml) and tebuconazole (0.0782–5 µg/ml) dissolved in dimethyl sulfoxide (DMSO). The colony diameter was measured in two perpendicular directions and values recorded in millimeters. EC_50_ values were calculated as described previously [16]. EC_50_ values of wild type and complemented strains were analyzed by the Mann–Whitney *U*-test.

### Microscopy

Spores of wild type and a GFP-expressing strain were germinated in cleared V8 juice (10%) and examined 3 days later using a Leica DM6000 microscope with an attached digital camera.

### Plant inoculations and pathogenicity testing

*Brassica napus* cultivar Westar was grown in soil in growth cabinets. Two weeks after sowing the seed, pycnidiospore suspensions (10^6^ spores/ml) from the *L. maculans* strains were placed as 10 µl drops onto wounded cotyledons. Lesions were scored on a 0–9 scale, as previously described [26], 11–14 days later.

## Results

### Development of a positive selection system in *L. maculans* based on resistance to iprodione due to mutations in the *hos1* gene

Wild type strains of *L. maculans* developed resistance to iprodione readily when mycelial plugs were inoculated on V8 juice agar medium supplemented with iprodione at concentrations up to 10 µg/ml. The fungicide at first inhibited growth, and then after several days a section of the mycelial plug initiated growth. These sectors were cultured and purified as single spore isolates.

**Fig. 1:**
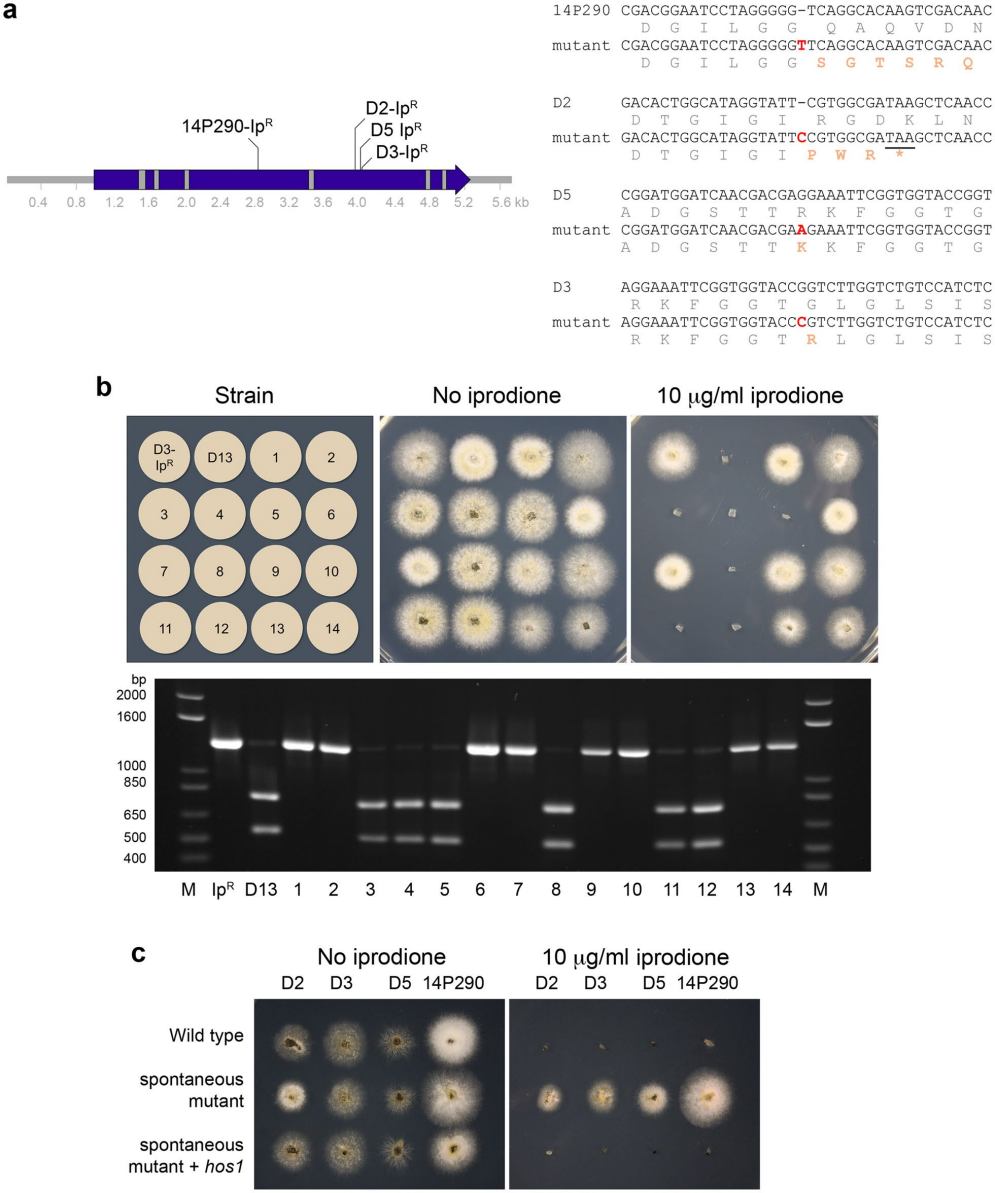
Spontaneous iprodione resistance occurs through mutation of the *hos1* gene. **a** Positions and nature of spontaneous mutations in iprodione resistant strains from four wild type strains relative to the exon (blue) and intron (grey) structure of the *hos1* gene. The sequence alignments are of the wild type and mutant strains, with the nucleotide mutations that occurred in the four strains in red bold, and the predicted amino acid sequences underneath. The mutations in strains 14P290 and D2 cause frame shifts (the stop codon in the D2 mutant is underlined, in 14P290 the new stop codon is beyond the sequence shown). The mutations in M1 and D3 cause amino acid substitutions in residues that are invariant across *hos1* homologs in the fungi. **b** A mutation in *hos1* co-segregates with iprodione resistance. Growth of two parents and 14 progeny (from 28 total) of a D13 × D3-Ip^R^ cross between the parents on PDA with or without iprodione. The alleles of *hos1* were assessed by PCR from genomic DNA of the two parents and 14 progeny from a cross between them, and subsequent digestion with AgeI restriction enzyme. M is the Invitrogen 1 kb + ladder. **c** Complementation of iprodione resistance back to sensitivity by the wild type *hos1* gene. Mycelial plugs were inoculated onto PDA medium with or without iprodione (10 μg/ml) and cultured 4 days. The strains are four wild type strains, four spontaneous mutants derived from them, and the four strains whereby the wild type copy of *hos1* was transformed into the mutants. The wild type copy of *hos1* returned the strains to the wild type sensitive phenotype

A single homolog, named *hos1*, of the osmosensing histidine kinase is present in *L. maculans* as assessed by BLAST analysis of the genome sequence [5]. Amplification of the *hos1* gene and sequencing revealed mutations in the gene in iprodione resistant mutants, all of which are predicted to cause a loss-of-function (Fig. 1a). Two mutants, one arising from strain D2 (referred to as D2-Ip^R^) and the other from strain 14P290 (P290-Ip^R^), cause frame shifts in the reading frame, leading to the introduction of premature stop codons. The mutations that occurred in the mutants arising from strains D5 (D5-Ip^R^) and D3 (D3-Ip^R^) cause amino acid substitutions (R923K and G929R, respectively). These two amino acid residues are highly conserved in homologs of the histidine kinase, because BLAST analysis of the fungal genomes available through the MycoCosm Portal of the Joint Genome Institute [27] revealed that both residues are invariant in all fungal species, including those of the early diverging lineages commonly termed the chytrids and zygomycetes.

Two methods were used to confirm that mutations in the *hos1* gene caused the resistance to iprodione. The first approach was to analyze the segregation of traits and genotypes in progeny from a cross. Resistant strain D3-Ip^R^ was crossed with sensitive strain D13, and 28 progeny obtained. The strains were scored for growth on medium containing iprodione and genotyped for the *hos1* allele by PCR–RFLP. The AgeI restriction enzyme recognition site (ACCGGT) of the wild type strain is lost in strain D3-Ip^R^ due to a base pair substitution (ACCCGT), as underlined. Nine progeny were sensitive to iprodione and 19 were resistant, which is not statistically different from the expected 1:1 ratio based on a χ^2^-test. The AgeI cut site polymorphism in *hos1* co-segregated with the iprodione sensitive or resistant phenotype (Fig. 1b). The second approach was complementation of the mutant phenotype. A wild type copy of the *hos1* gene was amplified and cloned into a plasmid that confers resistance to G418 for selection when the T-DNA is transformed into *L. maculans*. This construct was transformed into four strains that were iprodione resistant (i.e. D2-Ip^R^, D3-Ip^R^, D5-Ip^R^ and 14P290-Ip^R^). Transformants were tested on medium containing iprodione. Reintroducing the wild type copy of *hos1* into the strains caused them to become sensitive once again to iprodione, indicating that the mutations identified in *hos1* were the cause of the resistance to this antifungal agent (Fig. 1c).

A quantitative assay was used to measure the level of resistance to iprodione, a second dicarboximide chemical, procymidone, and an azole, tebuconazole, in four sets of strains (Table 2). The minimum inhibitory concentration was between 1 and 2 µg/ml for the wild type strains for the dicarboximides. The strains derived from selection on iprodione were not inhibited with these chemicals at concentrations up to 50 µg/ml. Complementation of the strains with the wild type copy of *hos1* restored the EC_50_ values to close to those seen in the wild type parents. In contrast, resistance to the unrelated molecule, tebuconazole (FRAC group 3), was not altered. Analysis of the results revealed no significant differences between wild type and restored strains on iprodione (*p* = 0.200–1.00), procymidone (*p* = 0.057–0.686) and tebuconazole (*p* = 0.333–1.00).

**Table 2:** Half maximal effective concentration (EC_50_) measurements of fungicide action for strains of *L. maculans*

Strain	Iprodione EC_50_ (μg/ml)		Procymidone EC_50_ (μg/ml)		Tebuconazole EC_50_ (μg/ml)	
	Average	SD	Average	SD	Average	SD
P290	1.25	0.09	1.75	0.04	0.29	0.01
P290-Ip^R^	> 50		> 50		0.53	0.01
P290-Ip^R^ + *hos1*	1.32	0.09	1.70	0.01	1.12	0.34
D2	1.10	0.22	1.94	0.12	0.40	0.14
D2-Ip^R^	> 50		> 50		0.39	0.02
D2-Ip^R^ + *hos1*	0.94	0.11	2.66	0.16	0.43	0.00
D3	1.21	0.06	1.84	0.13	0.91	0.01
D3-Ip^R^	> 50		> 50		0.51	0.02
D3-Ip^R^ + *hos1*	1.07	0.10	1.73	0.06	0.77	0.01
D5	0.43	0.27	1.57	0.20	0.50	0.03
D5-Ip^R^	> 50		> 50		0.10	0.05
D5-Ip^R^ + *hos1*	0.48	0.28	1.25	0.23	0.14	0.00

*SD* standard deviation

**Fig. 2:**
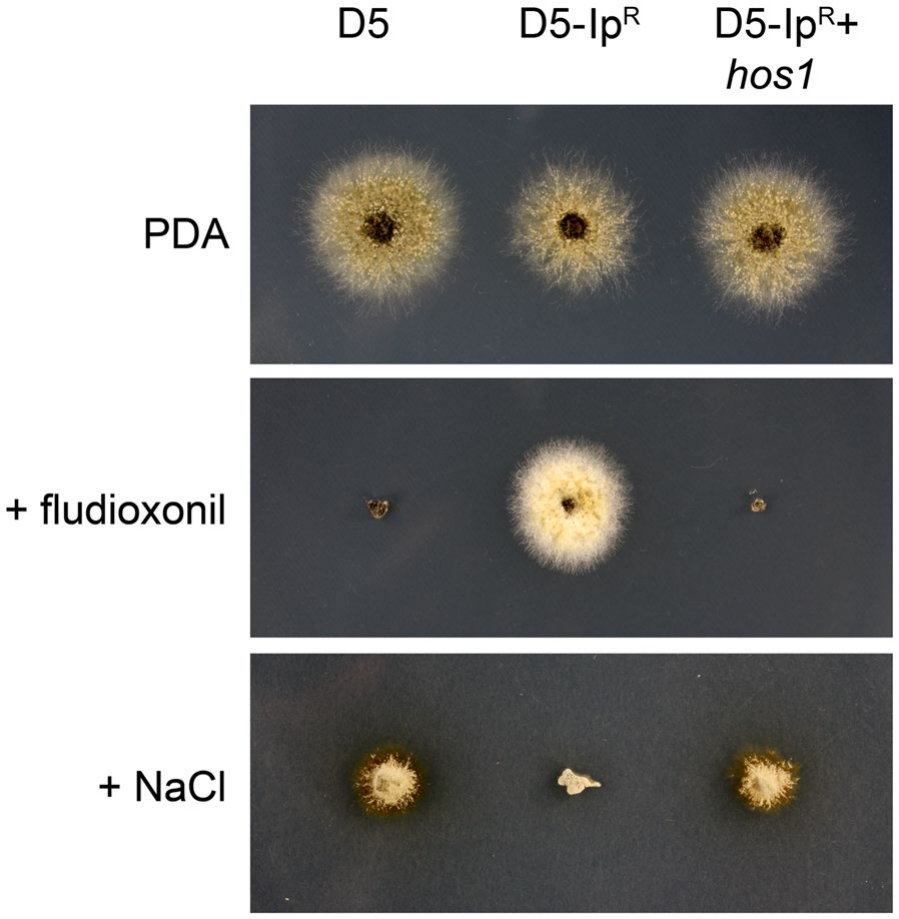
Mutation of *hos1* causes *L. maculans* to become resistant to the fungicide fludioxonil and more sensitive to NaCl. Strains D5, D5-Ip^R^ and D5-Ip^R^ + *hos1* and were cultured of PDA, and with PDA supplemented with fludioxonil (1 µg/ml) or NaCl (0.5 M)

Mutation of the HOG pathway components is also responsible for other phenotypes in fungi, the best known being changes in growth in the presence of the phenylpyrrole fungicide fludioxonil (FRAC group 12) and salt [12]. Using growth on plates, a wild type D5, *hos1* ^−^ mutant (D5-Ip^R^) and a + *hos1* complemented strain (D5-Ip^R^ + *hos1*) were examined for these properties. As has been reported for other fungi, mutation of *hos1* resulted in increased resistance to fludioxonil and an increased sensitivity to sodium chloride (Fig. 2).

**Fig. 3:**
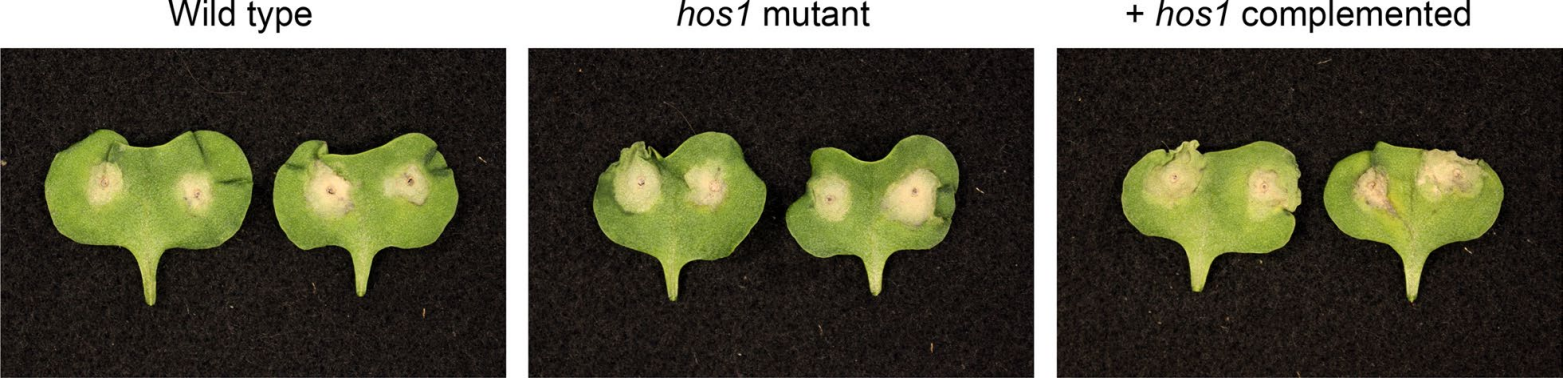
*L. maculans hos1* mutants are pathogenic on canola. Lesions on *B. napus* cv. Westar 11 days post inoculation caused by wild type isolate 14P290, a spontaneous mutation in *hos1* isolated on iprodione medium, and that strain complemented with a wild type copy of *hos1*

Growth *in planta* can be considered an environment of high stress to plant pathogenic fungi. Three *L. maculans* strains were inoculated onto wounded *B. napus* cotyledons, and lesion formation was examined over time. No difference in pathogenicity was observed between the three strains (average disease scores for wild type 6.14, *hos1* ^−^ mutant 6.03 and + *hos1* complementated 6.73; Fig. 3), indicating that *hos1* is not required for the ability of *L. maculans* to cause disease on canola.

### Development of a CRISPR/Cas9 gene disruption system for *L. maculans*

Identification of gene functions in *L. maculans* through targeted gene replacements has been inefficient due to low rates of homologous integration of constructs. The potential to use a positive selection system, i.e. growth on iprodione when *hos1* is mutated, was an impetus to use the *hos1* gene for the development of gene targeting methods, specifically through CRISPR-Cas9.

A versatile pair of plasmids (pLAU2 and pLAU53) was created for strong constitutive transcription of DNA sequences that are cloned into them. Both plasmids feature the 1001 bp prior to the start codon (the promoter) of the *act1* gene, encoding an actin subunit, and the terminator of *trp3* encoding anthranilate synthase of *L. maculans*. Actin is considered to be constitutively expressed and is commonly used as the reference gene in quantitative reverse transcriptase PCR experiments [21, 28, 29]. The plasmid includes a BglII site between the promoter and terminator into which genes or other DNA fragments can be cloned. To test if this promoter and terminator combination was able to drive protein production, the open reading frame for GFP was cloned into plasmid pLAU2, and the T-DNA transformed into wild type *L. maculans*. Fluorescence was abundant in spores and hyphae of transformants, indicating that the construct induces gene expression and yields high and stable protein synthesis (Additional file 3: Fig. S1).

**Fig. 4:**
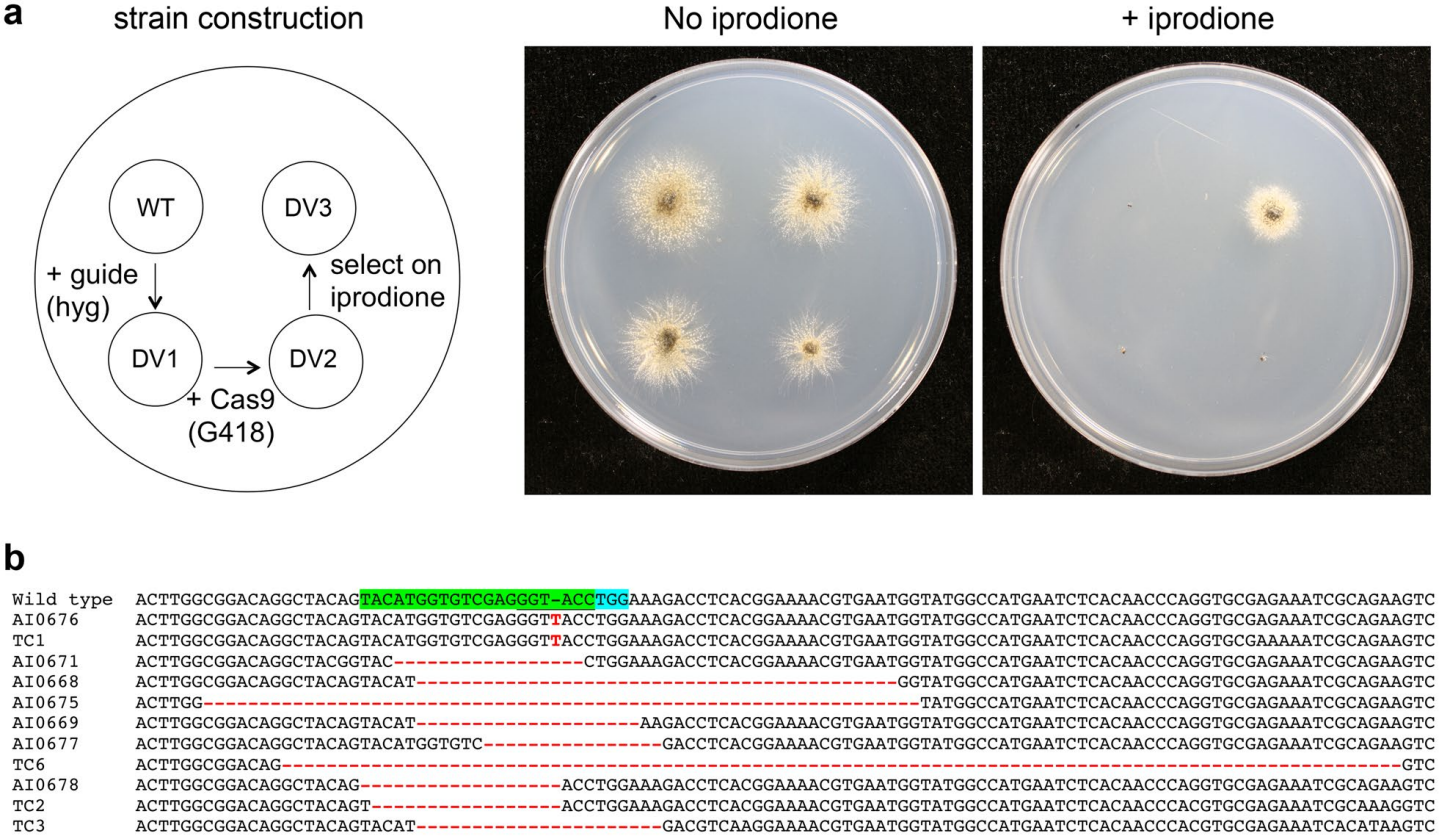
Development of the CRISPR/Cas9 system for targeted gene disruption in *L. maculans*. **a** Phenotype of transformants on plates with or without iprodione. Three strains derive from sequential modification of the wild type (WT) strain D5, first by transformation of the *hos1* guide RNA construct (strain DV1), then transformation of the Cas9 construct (strain DV2), and lastly by selection on iprodione (strain DV3). **b** Alignment of sequences of *hos1* from the wild type and 11 independently-created iprodione resistant mutants. On the wild type sequence the protospacer adjacent motif (PAM) is in blue highlight, region incorporated in the guide RNA in green highlight, and the KpnI restriction enzyme site used for screening is underlined. Changes in the sequence in the mutants are in red text

The open reading frame of the Cas9 endonuclease was amplified and cloned into both the pLAU2 or pLAU53 constructs. Similarly, a *hos1* RNA guide construct was cloned into both pLAU2 and pLAU53. As a consequence, different options were available for the order of transformation and selection of transformants. The T-DNAs were sequentially introduced into wild type strain D5, either as the *hos1* RNA guide first and Cas9 second (e.g. strains DV1 and DV2), or in the other order, and the transformants were plated onto media containing iprodione (5 µg/ml) to isolate resistant strains (Fig. 4a).

Iprodione resistant strains were cultured as mycelia, genomic DNA isolated, and the region spanning the site for CRISPR-Cas9 induced mutation in the *hos1* gene amplified. Amplicons were digested with KpnI restriction enzyme and/or sequenced. All isolates derived from strains expressing both Cas9 and the *hos1* RNA guide had mutations in this region (Fig. 4b). Most mutations were either the insertion of an additional nucleotide, or the deletion of one or several nucleotides (Fig. 4b). One strain, which is not illustrated in Fig. 4b due to the size of the DNA sequence, had a tandem duplication of 69 bp (acctggaaagacctcacggaaaacgtgaatggtatggccatgaatctcacaacccaggtgcgagaaatc). In all cases the mutations in *hos1* were near the region of the genome where the guide RNA would target Cas9, indicating that they were derived from inaccurate repair of DNA damage by the endonuclease. The types of mutations also often differed from those found in the spontaneous mutants as many featured large deletions, compared to single nucleotide substitutions or insertions.

The pathogenicity of strains from one set of gene manipulations were tested by inoculating *B. napus* cotyledons. The three strains derived from the wild type produced lesions like the wild type isolate (Additional file 4: Fig. S2). This indicates that the introduction of Cas9 or a guide RNA into *L. maculans* does not impact its pathogenicity.

### Improvements to mutation by CRISPR/Cas9

**Fig. 5:**
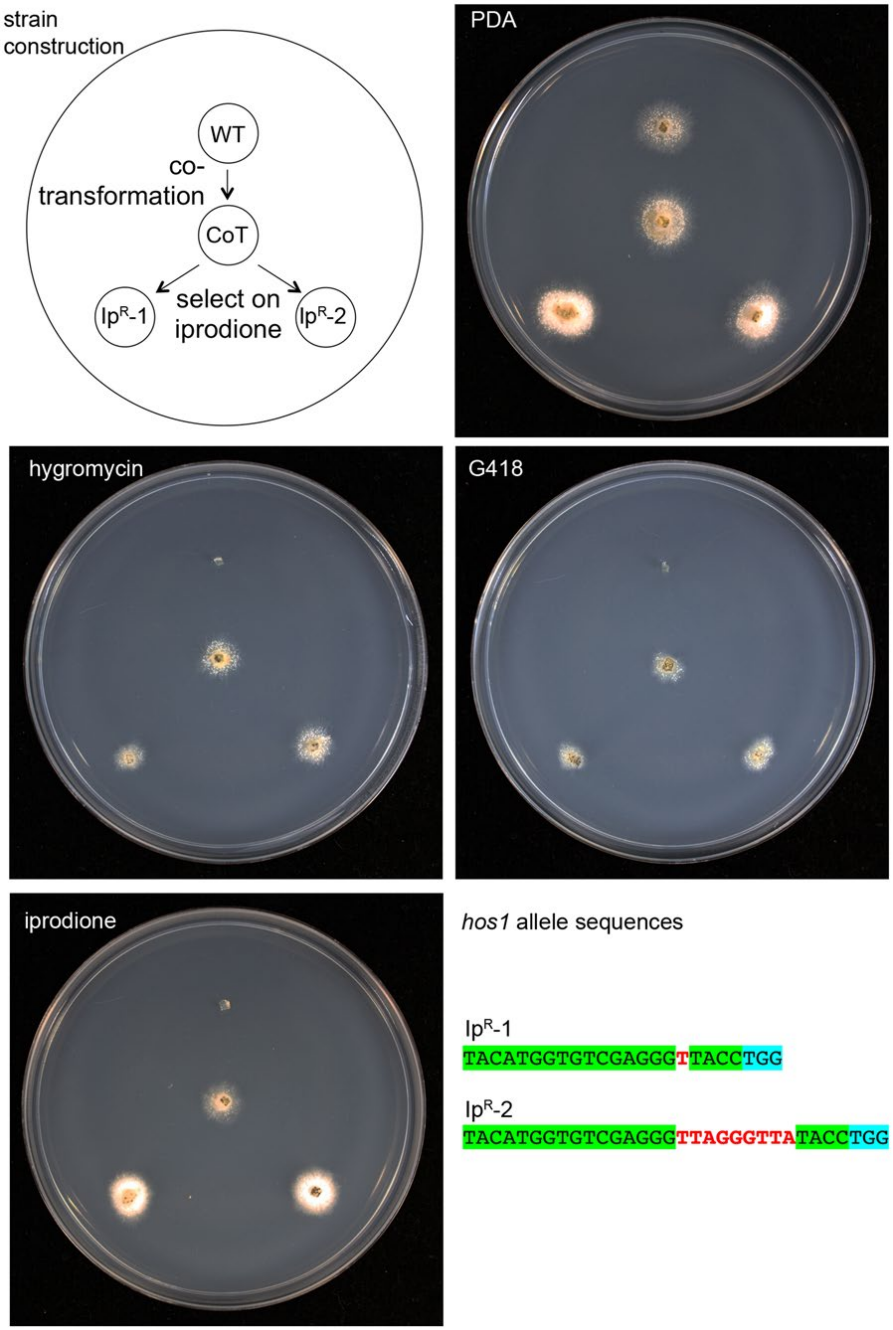
Co-transformation of Cas9 and the CRISPR guide RNA constructs into *L. maculans* by *Agrobacterium*-mediated transformation. Wild type strain D13 was co-transformed with T-DNAs from both constructs, and selected on media containing both hygromycin and G418. One transformant (D13-CoT) was cultured on medium containing iprodione. Two independent resistant strains were obtained, their DNA isolated, and the *hos1* mutations determined by amplification and sequencing. The region incorporated into the guide RNA is highlighted in green, and adjacent PAM site in blue highlight. The two iprodione resistant strains have additional nucleotides (red font) within the *hos1* gene

One disadvantage of the method to induce mutations by the CRISPR/Cas9 system developed here was the need to perform two rounds of transformation, and hence co-transformation was therefore tested. The wild type strain D13 was co-transformed with both the *hos1* RNA guide and Cas9 constructs, with simultaneous co-selection on media containing G418 and hygromycin. One double-drug resistant transformant (strain D13-CoT) was then cultured on medium containing iprodione, and two iprodione resistant isolates (D13-Ip^R^1 and D13-Ip^R^2) characterized by sequencing the *hos1* region (Fig. 5). Both strains have mutations caused by additional base pairs that can be attributed to the CRISPR-Cas9 system. While iprodione is generally considered non-mutagenic, the proportion of iprodione resistant spores were compared between the wild type D13 and the D13-CoT strains by culturing these in the absence of iprodione and then plating onto media with or without the fungicide. While the proportion of spores resistant to iprodione was less than 1 in 10,000 for the wild type, 54% of spores were resistant from the strain carrying the Cas9 endonuclease and *hos1*-guide construct.

To eliminate the requirement to order synthesized DNA fragments, which have to be cloned due to the complexity in secondary structure (Thermo Fisher Scientific), two plasmids were created that have the regions for the HDV ribozyme and Cas9-binding RNA. This new plasmid system was then used to target the first *Avr* gene identified in *L. maculans*, *AvrLm1*. The hammerhead ribozyme and a region to target the *AvrLm1* gene was synthesized as an 101 nucleotide oligonucleotide, amplified by PCR and cloned into the XhoI site of plasmid pMAI75. Cas9 was transformed into wild type strain v23.1.3, and then the guide RNA construct to mutate *AvrLm1* was transformed into this strain. Genomic DNA isolated from the resulting transformants was used as the templates to amplify a fragment of *AvrLm1*, and amplicons then cut with NlaIII restriction enzyme. Two strains without the NlaIII site were obtained and *AvrLm1* was amplified and sequenced from them. The mutant alleles had a single additional base pair or a 27 bp deletion (Additional file 5: Fig. S3). Thus, CRISPR-Cas9 can be used to isolate strains with mutations in genes, without a strong selection system as used for *hos1*.

## Discussion

Resistance to fungicides is a major problem in many areas of disease management, especially in agriculture with the high levels of antifungals applied to ensure maximum yield returns. For example, currently in Australia canola growers may apply triazole fungicides three times during a season, including as a seed dressing, in combination with fertilizer and as a foliar spray [1]. Stubble is retained in the field after harvest, and the subsequent crop in the same field may receive fungicide treatments, thereby inadvertently causing additional exposure to the *L. maculans* populations while growing as a saprophyte in the canola stubble.

Resistance to iprodione and other dicarboximide fungicides and the underlying mechanisms have been well characterized in other plant pathogenic fungi, such as *Botrytis cinerea* [30–32]. The impact of mutation of *hos1* in plant pathogens varies, in some cases impacting pathogenicity and in others having no effect [16]. Hallmarks of impairing the HOG pathway, i.e. increased resistance to fludioxonil and sensitivity to salt, occur upon mutation of the *hos1* gene in *L. maculans,* but the important ability to cause disease on plants is not. These results indicate that while iprodione is initially effective in vitro, resistance emerges easily, and these strains are still pathogenic on canola, potentially in part explaining the limited efficacy of iprodione in field conditions. Studies in other plant pathogenic fungi show differing results in terms of the contribution of the histidine kinase to pathogenicity. Similar to *L. maculans*, disruption of the gene does not impair pathogenicity in *Alternaria alternata*, *Parastagonospora nodorum* and *Pyricularia oryzae* [16, 33, 34]. In contrast, the homolog is required for pathogenicity in *Botrytis cinerea*, *Fusarium oxysporum*, *Monilinia fructicola*, *Sclerotinia sclerotiorum* and *Ustilago maydis* [35–39]. Deletion strains in *Alternaria longipes* form larger lesions on *Nicotiana tabacum* than the wild type [40]. There is ambiguity about the role of the gene in *A. brassicicola* with isolates with point mutations having wild type pathogenicity, while a deletion allele, albeit analyzed in a large scale study, being less pathogenic [41, 42]. The histidine kinase contributes to the virulence of the human pathogens *Candida albicans* and *Cryptococcus neoformans* [43, 44].

*Leptosphaeria maculans* continues to be a recurrent disease of oilseed Brassicas around the world because the factors that this fungus produces to cause disease are mostly unknown. One challenge with finding new ways to combat *L. maculans* is that identifying gene functions has been technically challenging. Gene disruption in *L. maculans* is inefficient, with only nine genes disrupted as reported in the literature [6–11]. Constructs require large amounts of DNA for targeting by homologous recombination and even then the proportion of gene deletion events versus ectopic integration of the constructs is low, e.g. an efficiency of just one knock out from > 450 transformants screened [6]. Advances in improving the proportion of targeted gene replacements versus ectopic integrations have been made, including the use of a counter selection system against ectopic insertion events [9] or using the selectable marker split into two pieces [11]. However, isolating the large DNA fragments needed and/or the cloning into suitable vectors imposes limitations to the efficiency of created targeted mutations. For this reason, alternative methods to disrupt genes are needed for *L. maculans*, and the method employing CRISPR-Cas9 was explored.

CRISPR/Cas is a combination of an endonuclease that is guided to a specific site in a genome using an RNA molecule, found in Bacteria and Archaea for recognition of parasitic DNA elements and their specific cleavage. Modified for use in other organisms, its ability to make specific double-stranded breaks in DNA that are then inaccurately repaired to induce small mutations, such as within genes, is on the cusp of revolutionizing methods for gene functional studies, including in fungal species that have until now been difficult to manipulate genetically.

Here we used iprodione resistance due to mutation of the *hos1* gene as an easy screening tool to develop CRISPR/Cas9 for *L. maculans*. Iprodione resistant strains derived from strains expressing Cas9 and a guide RNA targeting *hos1* all had mutations at the place within *hos1* where the endonuclease would cut the DNA. As proof-of-function that the method could work on other genes, the first avirulence gene that was identified in this fungus, *AvrLm1*, was disrupted [45]. Targeting these effectors, all found to date to lie within distinctive large regions of AT-rich and highly repetitive DNA [5, 46], has not been possible using homologous recombination. Curiously, the avirulence profile of these strains did not change as predicted (data not shown), and will require additional experiments to understand what is emerging in *L. maculans* as complex multigene sets of interactions between fungal avirulence genes and plant resistance genes [46, 47].

In the first iteration of CRISPR/Cas9 for *L. maculans*, two rounds of transformation were used to separately introduce the guide RNA and Cas9 expression constructs into the fungus. After seeking a suitable promoter for regulation by RNA polymerase III in *L. maculans* without success, the dual ribozyme system to process the guide RNA when expressed from an RNA polymerase II promoter was used. This dual ribozyme approach was developed for plant transformation [48], and has recently also been employed in *Aspergillus* spp. [49], the basidiomycete human pathogen *Cryptococcus neoformans* [50] and the ascomycete plant pathogen *Alternaria alternata* [51]. The disadvantage of using ribozymes for processing the guide RNA is that they add size to the constructs. To alleviate this issue, we created a vector such that just one of the ribozymes and the RNA fragment to target Cas9 to the gene to be mutated are synthesized: the hammerhead ribozyme requires folding with part of the target RNA and hence there is a requirement for long ~ 100 nucleotide oligonucleotides. The current method, although involving two transformation steps or co-transformation of both constructs, is suitable for making targeted mutations in genes, and has been tested in multiple wild type isolates. We have mutated more than 24 other genes in *L. maculans* to date (unpublished data). Potential refinements to the method in the near future will likely make it even more effective as a mutational tool to discover gene functions in *L. maculans*.

## Additional files

**Additional file 1: Table S1.** Oligonucleotide primers used in this study.

**Additional file 2.** Sequence of the guide RNA constructs that were synthesized. Colors of the nucleotides infer different purposes. Blue, primer binding sites for amplification and cloning into plasmids; grey, ribozymes; red, 20 nucleotides specific to *hos1*; purple, guide RNA; black, stop codon for *trp3*. Underlined nucleotides will base pair in the hammerhead ribozyme. Bold is the XhoI restriction enzyme site.

**Additional file 3: Fig. S1.** Constitutive expression of genes using the actin regulatory sequences. The *L. maculans* actin (*act1*) promoter and 5′ UTR were cloned to allow expression of adjacent genes. In this case, GFP was fused to this region, and the construct transformed into *L. maculans*. Spores were germinated for 3 days in 10% cleared V8 juice media. Bar = 50 µm.

**Additional file 4: Fig. S2.** Expression of the CRISPR components or mutation of *hos1* does not impair pathogenicity on plants. Cotyledons of *B. napus* cv. Westar were inoculated with four strains, and lesions measured 14 days later. The wild type isolate D5 was sequentially transformed with the *hos1* guide RNA construct (to create strain DV1) and the Cas9 construct (DV2), and then plated on iprodione to isolate a resistant strain (DV3).

**Additional file 5: Fig. S3.** Targeted mutation of the *AvrLm1* gene in *L. maculans* by CRISPR-Cas9. Alignment of the coding region of *AvrLm1* from wild type and two mutant alleles. On the wild type sequence the PAM is in blue highlight, region incorporated in the guide RNA in green, and the NlaIII restriction enzyme site used for screening is underlined. The differences in sequence in the two mutants are in red, as an extra A nucleotide or a deletion of 27 nucleotides.
