# Supplementary material for: Spontaneous and CRISPR/Cas9-induced mutation of the osmosensor histidine kinase of the canola pathogen Leptosphaeria maculans
Source: Fungal Biol Biotechnol. 2017 Dec 16;4:12. doi: 10.1186/s40694-017-0043-0 (PMC5732519; doi:10.1186/s40694-017-0043-0)
Supplement: Supplementary file 1 — Additional file 1: Table S1. Oligonucleotide primers used in this study. [file 40694_2017_43_MOESM1_ESM.pdf]

Name	Sequence (5´-3´)	Purpose
AU1	AGATCTGAGGGATGTGACTATGAGC	*act1* promoter
AU2	AGATCTGAGGGATGTGACTATGAGC	
AU5	AGATCTGAGGGATGTGACTATGAGC	*trp3* terminator
AU6	AGATCTGAGGGATGTGACTATGAGC	
AU28	AGATCTGAGGGATGTGACTATGAGC	GFP
AU31	AGATCTGAGGGATGTGACTATGAGC	
MAI0206	TAGGCCTCTGCAGGTCGACTCCTACTATGTATGCACTTGG	Complementation with wild type copy of *hos1*
MAI0207	TCCCAGAATTCTTAATTAAGATTGACACCCTTCGCACAAC	
MAI0014	GAATTCTGGGATTGCCCCTCGATGC	*act1* promoter
MAI0015	TCCATCTTGTTCAATCATGTTTGATTGATTAGG	
MAI0016	CTTGACGAGTTCTTCTGAGGTATGCAGACTTTGGC	*act1* terminator
MAI0017	GAATTCCTACTGAACGTTATGACG	
ALID0835	ATGATTGAACAAGATGGATTGC	G418 phosphotransferase
ALID0836	TCAGAAGAACTCGTCAAGAAGG	
MAI0018	CTGCAGCTGGGATTGCCCCTCGATGC	*act1* promoter
MAI0024	GAGTTCAGGCTTTTTCATGTTTGATTGATTAGG	
MAI0020	CCGAGGGCAAAGGAATAGGGTATGCAGACTTTGGC	*act1* terminator
MAI0021	GGTACCTACTGAACGTTATGAC	
MAI0022	ATGAAAAAGCCTGAACTCAC	Hygromycin phosphotransferase
MAI0023	CTATTCCTTTGCCCTCGGAC	
MAI0218	GCACAGAGGGAAGGCTTG	Part of *hos1*
MAI0223	ACTGAAAAACGTACTGGTCC	
MAI0220	ATGGAGGGCAAATTTACG	Part of *hos1*
MAI0224	TATGCAATGAGTTATCGGCG	
MAI0376	CGAGTGGTCCGAGACCAG	
MAI0225	GAAACCTAATCAATCAACATGGATTACAAGGACCAC	Cas9
MAI0226	GCTCATAGTCACATCCCTCACTTCTTCTTCTTCGCCTG	
MAI0228	GAAACCTAATCAATCAACCATGTACTGATGAGTCCG	double ribozyme-*hos1*-CRISPR guide RNA
MAI0229	GCTCATAGTCACATCCCTCAGTCCCATTCGCCATGCCG	
MAI0336	GAAACCTAATCAATCAACCTTCACCTGATGAGTCCGTGAGGACGAAACGAGTAAGCTCGTCGTGAAGGTCCGTTTCATGTGGTTTTAGAGCTAGAAATAGC	*AvrLm1* guide + hammerhead ribozyme
MAI0309	ACCTCTAATCGAAACCTAATCAATCAAC	Amplify *AvrLm1* guide + hammerhead ribozyme
MAI0310	ATTTTAACTTGCTATTTCTAGCTCTAAAAC	
MAI0353	CTATCAACAGCTCTTGCAGC	Screening *AvrLm1* CRISPR mutation
MAI0354	AGTTCAACATTCGCTTTGCC	
