# Supplementary material for: Spontaneous and CRISPR/Cas9-induced mutation of the osmosensor histidine kinase of the canola pathogen Leptosphaeria maculans
Source: Fungal Biol Biotechnol. 2017 Dec 16;4:12. doi: 10.1186/s40694-017-0043-0 (PMC5732519; doi:10.1186/s40694-017-0043-0)
Supplement: Supplementary file 2 — Additional file 2. Sequence of the guide RNA constructs that were synthesized. Colors of the nucleotides infer different purposes. Blue, primer binding sites for amplification and cloning into plasmids; grey, ribozymes; red, 20 nucleotides specific to hos1; purple, guide RNA; black, stop codon for trp3. Underlined nucleotides will base pair in the hammerhead ribozyme. Bold is the XhoI restriction enzyme site. [file 40694_2017_43_MOESM2_ESM.pdf]

Hammerhead ribozyme - *hos1* - RNA guide – HDV ribozyme

GAAACCTAATCAATCAACCATGTACTGATGAGTCCGTGAGGACGAAACGAGTAAGC
TCGTCTACATGGTGTCGAGGGTACCGTTTTAGAGCTAGAAATAGCAAGTTAAAATAA
GGCTAGTCCGTTATCAACTTGAAAAAGTGGCACCGAGTCGGTGCTTTTGGCCGGCAT
GGTCCCAGCCTCCTCGCTGGCGCCGGCTGGGCAACATGCTTCGGCATGGCGAATGG
GACTGAGGGATGTGACTATGAGC

RNA guide - HDV ribozyme

GAAACCTAATCAATCAAC**TCGAG**TTTTAGAGCTAGAAATAGCAAGTTAAAATAAGG
CTAGTCCGTTATCAACTTGAAAAAGTGGCACCGAGTCGGTGCTTTTGGCCGGCATGG
TCCCAGCCTCCTCGCTGGCGCCGGCTGGGCAACATGCTTCGGCATGGCGAATGGGAC
TGAGGGATGTGACTATGAGC
