# Supplementary figures and images for: Spontaneous and CRISPR/Cas9-induced mutation of the osmosensor histidine kinase of the canola pathogen Leptosphaeria maculans
Source: Fungal Biol Biotechnol. 2017 Dec 16;4:12. doi: 10.1186/s40694-017-0043-0 (PMC5732519; doi:10.1186/s40694-017-0043-0)

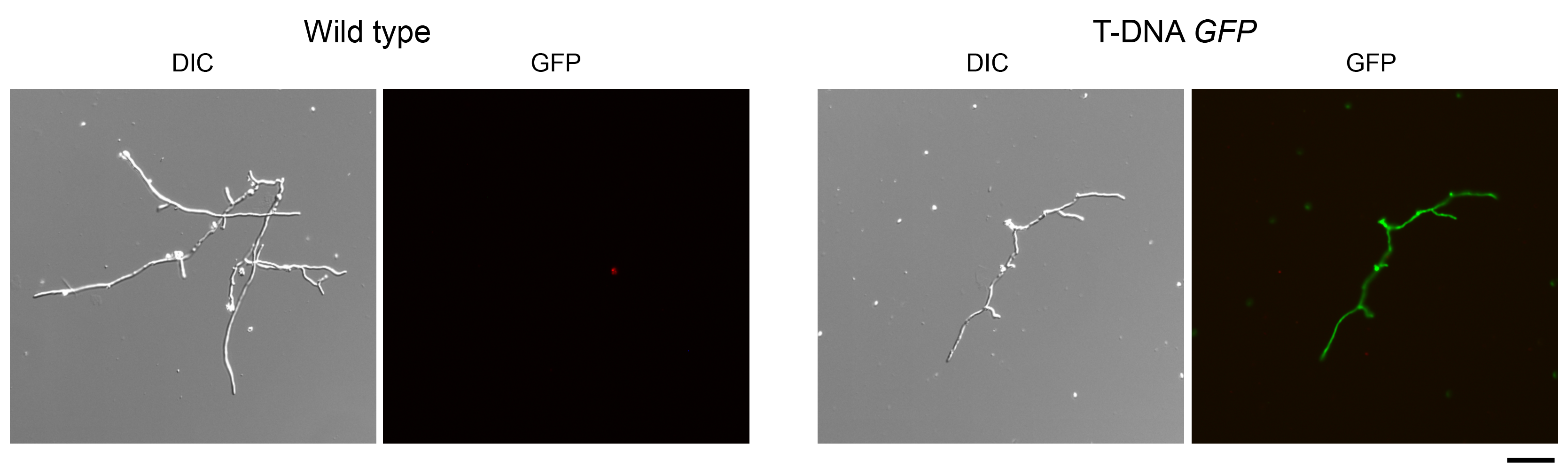

Supplement: Supplementary file 3 — Additional file 3: Fig. S1. Constitutive expression of genes using the actin regulatory sequences. The L. maculans actin (act1) promoter and 5′ UTR were cloned to allow expression of adjacent genes. In this case, GFP was fused to this region, and the construct transformed into L. maculans. Spores were germinated for 3 days in 10% cleared V8 juice media. Bar = 50 µm. [file 40694_2017_43_MOESM3_ESM.tif]

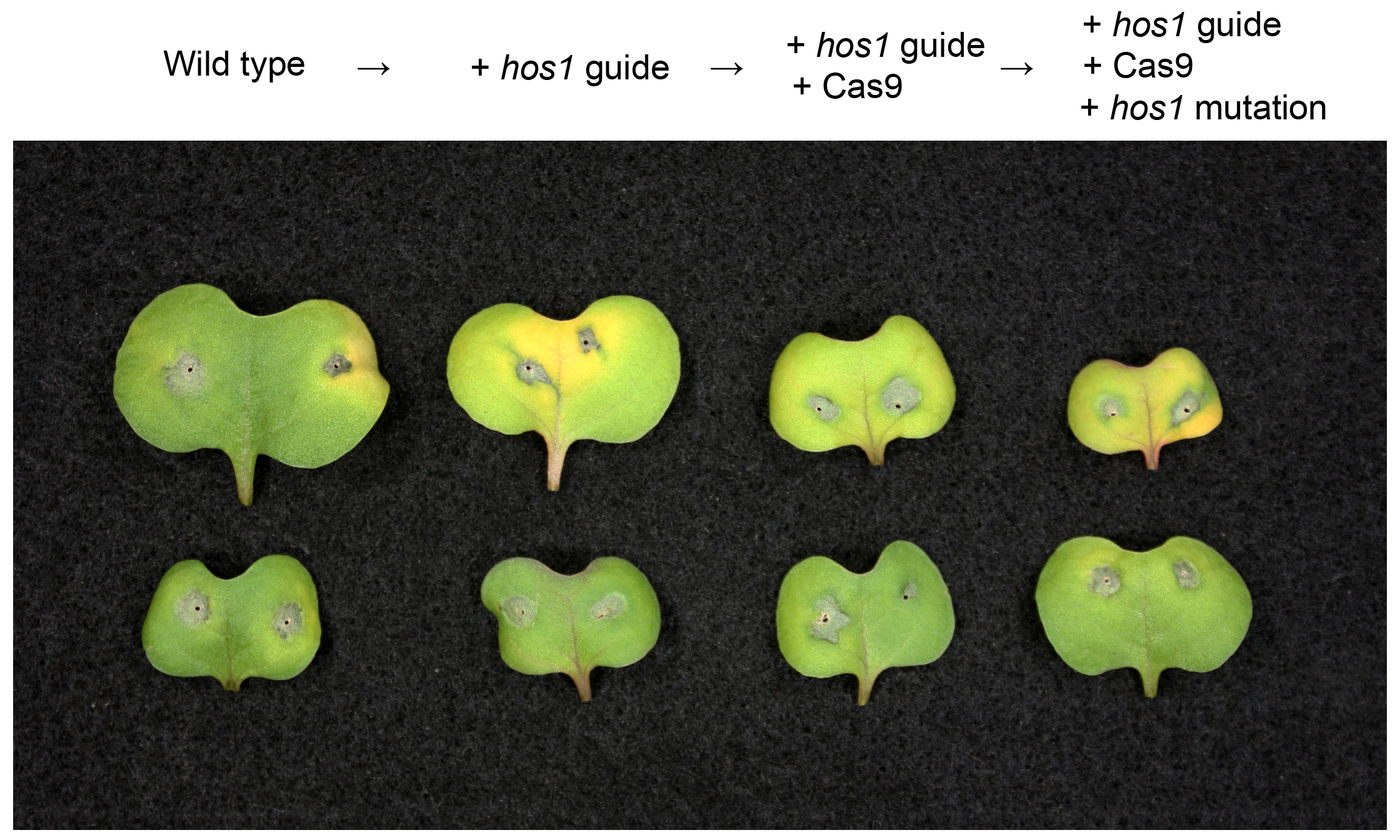

Supplement: Supplementary file 4 — Additional file 4: Fig. S2. Expression of the CRISPR components or mutation of hos1 does not impair pathogenicity on plants. Cotyledons of B. napus cv. Westar were inoculated with four strains, and lesions measured 14 days later. The wild type isolate D5 was sequentially transformed with the hos1 guide RNA construct (to create strain DV1) and the Cas9 construct (DV2), and then plated on iprodione to isolate a resistant strain (DV3). [file 40694_2017_43_MOESM4_ESM.tif]

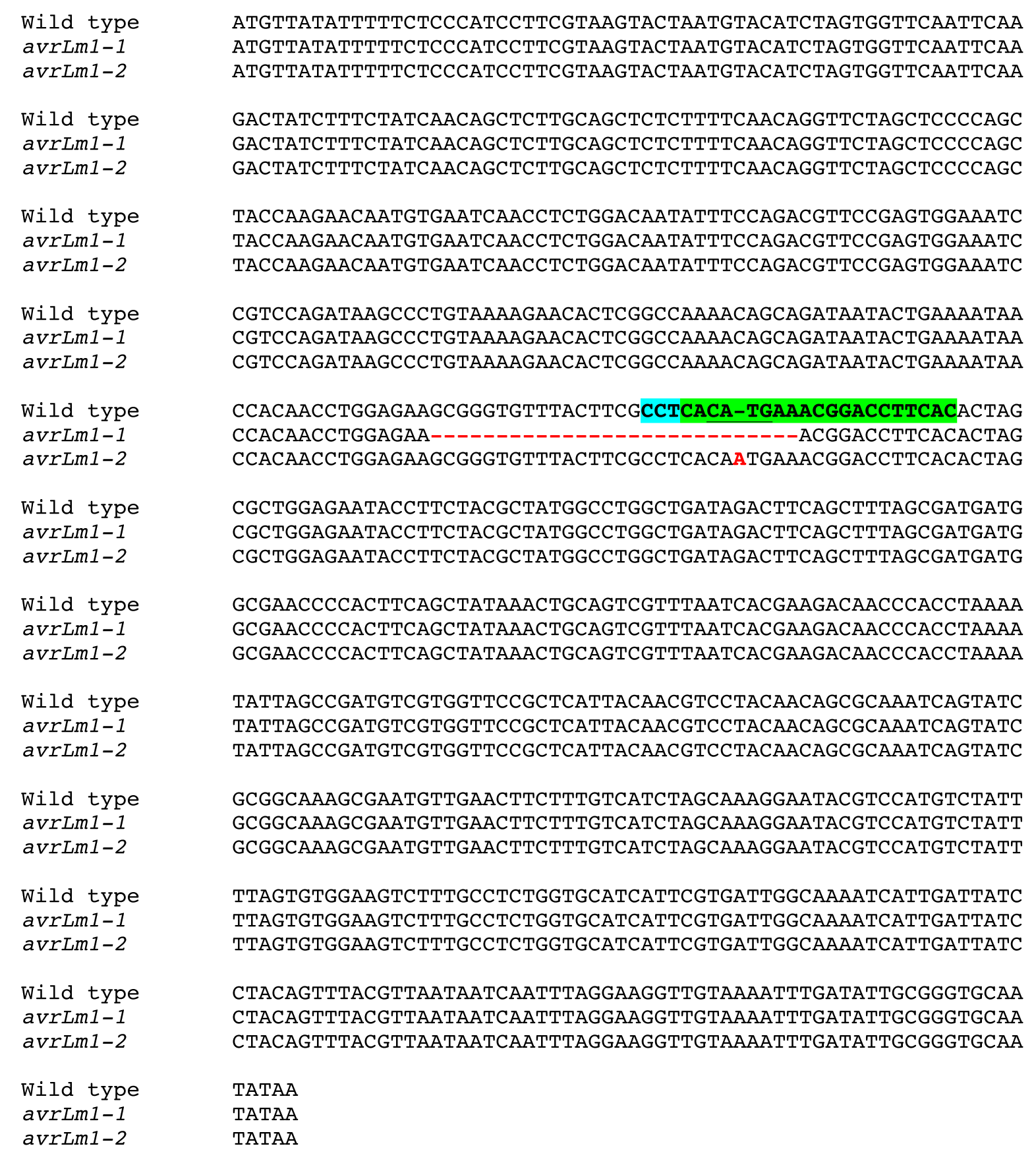

Supplement: Supplementary file 5 — Additional file 5: Fig. S3. Targeted mutation of the AvrLm1 gene in L. maculans by CRISPR-Cas9. Alignment of the coding region of AvrLm1 from wild type and two mutant alleles. On the wild type sequence the PAM is in blue highlight, region incorporated in the guide RNA in green, and the NlaIII restriction enzyme site used for screening is underlined. The differences in sequence in the two mutants are in red, as an extra A nucleotide or a deletion of 27 nucleotides. [file 40694_2017_43_MOESM5_ESM.tif]
